# Gains in Disability-Free Life Expectancy From Elimination of Diseases and Injuries in Japan

**DOI:** 10.2188/jea.JE20110112

**Published:** 2012-05-05

**Authors:** Shuji Hashimoto, Miyuki Kawado, Hiroya Yamada, Rumi Seko, Yoshitaka Murakami, Masayuki Hayashi, Masahiro Kato, Tatsuya Noda, Toshiyuki Ojima, Masato Nagai, Ichiro Tsuji

**Affiliations:** 1 Department of Hygiene, Fujita Health University School of Medicine, Toyoake, Japan; 2 Faculty of Nursing, Fujita Health University School of Health Sciences, Toyoake, Japan; 3 Department of Medical Statistics, Shiga University of Medical Science, Otsu, Japan; 4 Department of Information Science, Fukushima Medical University School of Nursing, Fukushima, Japan; 5 Tsushima Public Health Center, Aichi Prefecture, Tsushima, Japan; 6 Department of Community Health and Preventive Medicine, Hamamatsu University School of Medicine, Hamamatsu, Japan; 7 Division of Epidemiology, Department of Public Health and Forensic Medicine, Tohoku University Graduate School of Medicine, Sendai, Japan

**Keywords:** disability-free life expectancy, healthy life expectancy, life expectancy, activities of daily living, health statistics

## Abstract

**Background:**

Although disability-free life expectancy has been investigated in Japan, gains from elimination of diseases and injuries have not been examined.

**Methods:**

We used data from the 2007 Japanese national health statistics to calculate the number of years with and without activity limitation that could be expected from eliminating 6 selected diseases and injuries.

**Results:**

At birth, the number of expected years of life without and with activity limitation was 70.8 and 8.4, respectively, in males and 74.2 and 11.8 in females. More than 1.0 expected years without activity limitation were gained from eliminating malignant neoplasms and cerebrovascular diseases; smaller gains were observed after eliminating other diseases and injuries. Elimination of cerebrovascular diseases, dementia, and fracture decreased expected years with activities of daily living (ADL) limitation, and elimination of shoulder lesions/low back pain decreased expected years with non-ADL limitation.

**Conclusions:**

Elimination of diseases and injuries increased expected years with and without activity limitation among Japanese, which suggests that improved prevention of those diseases and injuries—including cerebrovascular diseases and dementia—would result in longer disability-free life expectancy and fewer years of severe disability.

## INTRODUCTION

Improvement of disability-free life expectancy requires evaluation of the impact of diseases and injuries.^1^ Disability-free life expectancy gained from elimination of diseases and injuries was proposed as an indicator of disease burden and has been investigated in several countries.^1^^–^^6^

In Japan, life expectancy at birth is now the longest in the world, and gains in years of life due to elimination of causes of death are reported annually in official statistics.^7^^,^^8^ Recently, expected years of life with and without activity limitation have been studied, but gains from elimination of diseases and injuries have not yet been examined.^9^

In the present study, we used 2007 Japanese national health statistics data to calculate gains in years of life, with and without activity limitation, that would be expected if selected diseases and injuries were eliminated.

## METHODS

### Data

We used data from life tables, the population, and number of deaths in Japan in 2007.^8^^,^^10^^,^^11^ Data on activity status and disease status for persons living at home were obtained from the 2007 Comprehensive Survey of Living Conditions of the People on Health and Welfare, which was a self-administered questionnaire survey distributed to about 760 000 persons in households randomly selected nationwide.^12^ Data for patients admitted to hospitals and clinics were from the Patient Surveys of 2005 and 2008, which included information on more than 3 000 000 patients who visited hospitals and clinics randomly selected throughout Japan.^13^ Data for Japanese who were admitted to healthcare and welfare facilities for elderly requiring long-term care (hereafter, “residents of long-term elder care facilities”) were from the 2007 Survey of Institutions and Establishments for Long-term Care.^14^ Data from the 3 surveys were used with permission from the Ministry of Internal Affairs and Communications and the Ministry of Health, Labour and Welfare of Japan.

### Activity limitation

The activity status of persons living at home was evaluated using responses to the questions: “Is your daily life now affected by health problems?” and “How is it affected?”.^12^ The second question was for persons replying “Yes” to the first question. The responses to the second question were “activities of daily living (ADL) (rising, dressing/undressing, eating, bathing, etc),” “going out,” “work, housework, or schoolwork,” “physical exercise (including sports),” and “other.” We accordingly classified the responses into 3 levels of activity. A person replying “Yes” to the first question and “ADL” to the second was classified as having an ADL limitation. A person replying “Yes” to the first question but not “ADL” to the second was classified as having a non-ADL limitation. Respondents with other replies were classified as having no activity limitation. Inpatients in hospitals and clinics and residents of long-term elder care facilities were considered to have an ADL limitation.

### Disease status

We selected 6 diseases and injuries: malignant neoplasms (International Classification of Diseases, 10th Revision [ICD-10] code: C00–C97), ischemic heart disease (I20–I25), cerebrovascular diseases (I60–I69), dementia (F00–F03; G30), shoulder lesions/low back pain (M54.3–M54.5; M75), and fracture (S02, S12, S22, S32, S42, S52, S62, S72, S82, S92, T02, T08, T10, T12, T14.2).^11^^,^^13^

Disease status for persons living at home was evaluated using responses to the questions: “Do you now go to a hospital, clinic, or facility of Japanese traditional massage, acupuncture, moxibustion, or judo-orthopedics for diseases or injuries?” and “What are your diseases or injuries?”.^12^ The second question was for persons replying “Yes” to the first question. The responses to the second question were 39 diseases and injuries that were encompassed by the abovementioned 6 diseases and injuries, “other disease or injury”, and “unknown.” A person who indicated in the second question that they had any of the 6 diseases and injuries was classified as an outpatient with that disease or injury. For inpatients in hospitals and clinics and residents of long-term elder care facilities, the primary disease or injury was used to determine the presence or absence of the 6 diseases and injuries.^13^^,^^14^ Underlying cause of death was used in the analysis.^11^

### Calculation of gains in years with and without activity limitation expected from elimination of diseases and injuries

We calculated expected years of life with and without activity limitation that would be gained from eliminating each of the above 6 diseases and injuries in Japan in 2007. Gains were defined as years after elimination minus those years without disease elimination. The method used to calculate years with no disease elimination was equivalent to one used in a previous Japanese report analyzing the period 1995–2004.^9^ The previously used method for calculating years from elimination of a specific disease or injury is described below.^2^

A life table that eliminated deaths caused by disease was constructed using data on number of deaths and life tables without disease elimination.^8^^,^^11^^,^^15^ The probability of survival in age group *x* with the disease eliminated (*p_x_^e^*) was expressed using the probability without disease elimination (*p_x_*), the number of deaths (*D_x_*) from all diseases and injuries, and the number of deaths from the disease (*D_x_^e^*), as follows:

$$\ln(p_{x}{}^{e})=(1-D_{x}{}^{e}/D_{x})\ln(p_{x})$$

where ln is a natural logarithm function and the age groups are 0 to 4, 5 to 9,…, 80 to 84, and 85 years or older. Using Chiang’s life table method,^16^ the number of survivors (*l_x_^e^*) and the stationary population (*L_x_^e^*), the effect of eliminating a disease was calculated from the values of *p_x_^e^*.

We calculated 2007 sex- and age-specific prevalences of ADL limitation and non-ADL limitation after disease elimination. The prevalence of ADL limitation after eliminating a disease was based on the population after excluding outpatients with the disease and ADL limitations, inpatients with the disease in hospitals and clinics, and people with the disease who resided in long-term elder care facilities. The prevalence of non-ADL limitation after eliminating a disease was calculated from the population excluding outpatients with the disease and non-ADL limitation. The prevalence of inpatients in 2007 was estimated from those in 2005 and 2008 using linear interpolation, and other 2007 prevalences were derived from the abovementioned data.

Using the Sullivan method,^17^ we divided years of life in age group *x* (*e_x_^e^*) expected after eliminating a disease into those with and without activity limitation, as follows:

$$e_{x}{}^{e}=\Sigma \pi_{y}{}^{e}L_{y}{}^{e}/l_{x}{}^{e}+\Sigma (1-\pi_{y}{}^{e})L_{y}{}^{e}/l_{x}{}^{e}$$

where Σ represents the sum from age group *x* to the oldest age group in the age group of *y* and π*_y_^e^* is the age-specific prevalence of activity limitation after eliminating the disease. In addition, we divided the years with activity limitation expected after eliminating a disease into those due to ADL limitation and those due to non-ADL limitation.

## RESULTS

Tables 1 and 2 show death rates, prevalences, and proportions of selected diseases and injuries by age group in males and females, respectively. Malignant neoplasms, ischemic heart disease, and cerebrovascular diseases were associated with high death rates, whereas dementia, shoulder lesions/low back pain, and fracture were associated with low death rates. Among those aged 65 years or older, a large proportion of residents of long-term elder care facilities had cerebrovascular diseases and dementia and a large proportion of inpatients had cerebrovascular diseases. Among those aged 0 to 64 years and those aged 65 years or older, large proportions of outpatients had shoulder lesions/low back pain. Among outpatients with either dementia or fracture, the proportion of those with no limitation of activities was low; a high proportion of outpatients with dementia had an ADL limitation.

**Table 1.** Death rate, prevalence, and proportion of selected diseases and injuries by age group in males

	Death rate (per 100 000 population)	Prevalence (per 1000 population)			Proportion of outpatients (%)^c^		
	
		Residents admitted to facilities^a^	Inpatients^b^	Outpatients^c^	No limitation of activities	Non-ADL limitation	ADL limitation
Age 0–64 years							
All diseases and injuries	251.1	0.1	5.3	227.5	74.1	19.0	6.9
Malignant neoplasms	88.4	0.0	0.5	1.8	55.0	36.1	8.9
Ischemic heart disease	18.0	0.0	0.1	5.8	64.9	26.4	8.7
Cerebrovascular diseases	20.3	0.1	0.4	4.3	50.6	23.2	26.1
Dementia	0.1	0.0	0.0	0.2	32.6	38.6	28.8
Shoulder lesions/low back pain	0.0	0.0	0.0	26.7	69.9	22.8	7.3
Fracture	3.1	0.0	0.2	3.2	41.3	34.5	24.2
Age 65 years or older							
All diseases and injuries	4010.6	12.6	33.0	654.8	67.0	19.1	13.8
Malignant neoplasms	1361.9	0.3	4.9	12.6	45.6	31.2	23.1
Ischemic heart disease	274.8	0.2	0.7	58.7	53.0	29.3	17.7
Cerebrovascular diseases	436.7	5.0	6.1	44.9	41.8	20.9	37.3
Dementia	18.7	2.8	2.0	10.5	18.2	17.4	64.5
Shoulder lesions/low back pain	0.1	0.0	0.0	96.4	52.3	29.5	18.1
Fracture	12.8	0.2	1.1	6.4	23.8	35.2	41.0

ADL, activities of daily living.

^a^Healthcare and welfare facilities for elderly requiring long-term care.

^b^Inpatients in hospitals and clinics.

^c^Outpatients in hospitals, clinics, and facilities of Japanese traditional massage, acupuncture, moxibustion, and judo-orthopedics.

**Table 2.** Death rate, prevalence, and proportion of selected diseases and injuries by age group in females

	Death rate (per 100 000 population)	Prevalence (per 1000 population)			Proportion of outpatients (%)^c^		
	
		Residents admitted to facilities^a^	Inpatients^b^	Outpatients^c^	No limitation of activities	Non-ADL limitation	ADL limitation
Age 0–64 years							
All diseases and injuries	120.6	0.1	4.3	266.9	73.4	19.6	6.9
Malignant neoplasms	58.7	0.0	0.4	4.2	61.0	29.6	9.3
Ischemic heart disease	4.4	0.0	0.0	2.7	64.8	24.2	11.0
Cerebrovascular diseases	9.2	0.1	0.2	2.1	47.7	27.1	25.2
Dementia	0.1	0.0	0.0	0.2	36.0	25.2	38.8
Shoulder lesions/low back pain	0.0	0.0	0.0	44.9	72.0	21.0	7.0
Fracture	1.3	0.0	0.1	2.4	37.8	34.3	27.9
Age 65 years or older							
All diseases and injuries	2907.9	33.8	35.3	635.0	63.2	20.1	16.7
Malignant neoplasms	669.0	0.3	2.4	7.7	41.7	34.1	24.2
Ischemic heart disease	203.6	0.6	0.5	35.7	46.8	27.7	25.5
Cerebrovascular diseases	392.1	8.6	7.2	21.7	34.2	22.3	43.5
Dementia	30.7	10.5	3.4	14.7	18.7	17.4	63.9
Shoulder lesions/low back pain	0.0	0.1	0.1	137.9	50.8	28.3	20.9
Fracture	13.4	1.5	3.4	13.0	25.0	24.9	50.0

ADL, activities of daily living.

^a^Healthcare and welfare facilities for elderly requiring long-term care.

^b^Inpatients in hospitals and clinics.

^c^Outpatients in hospitals, clinics, and facilities of Japanese traditional massage, acupuncture, moxibustion, and judo-orthopedics.

Table 3 shows baseline years and gains in years, at birth, with and without activity limitation expected after eliminating the selected diseases and injuries. Life expectancy at birth was 79.2 years in males and 86.0 years in females. There were large gains in life expectancy from eliminating malignant neoplasms, ischemic heart disease, and cerebrovascular diseases (0.6–4.0 years) and very small gains from eliminating the other 3 diseases and injuries (0.0–0.1 years).

**Table 3.** Baseline and gains in years with and without activity limitation, at birth, expected from elimination of selected diseases and injuries

		Life expectancy at birth	Expected years at birth			

			Without activity limitation	With activity limitation	With non-ADL limitation	With ADL limitation
Males	At baseline	79.19	70.80	8.39	4.60	3.79
	Gains from elimination of					
	malignant neoplasms	4.00	2.78	1.22	0.48	0.75
	ischemic heart disease	0.72	0.70	0.02	−0.11	0.13
	cerebrovascular diseases	1.04	1.13	−0.09	0.10	−0.19
	dementia	0.03	0.17	−0.14	0.02	−0.16
	shoulder lesions/low back pain	0.00	0.59	−0.59	−0.51	−0.07
	fracture	0.10	0.26	−0.16	−0.07	−0.09

Females	At baseline	85.99	74.20	11.79	5.86	5.93
	Gains from elimination of					
	malignant neoplasms	2.98	1.96	1.02	0.28	0.74
	ischemic heart disease	0.56	0.42	0.14	−0.05	0.19
	cerebrovascular diseases	1.13	1.04	0.09	0.15	−0.06
	dementia	0.07	0.37	−0.30	0.06	−0.37
	shoulder lesions/low back pain	0.00	0.82	−0.82	−0.79	−0.04
	fracture	0.07	0.30	−0.23	−0.04	−0.19

ADL, activities of daily living.

The number of expected years without and with activity limitation was 70.8 and 8.4 years in males, respectively, and 74.2 and 11.8 years in females. Elimination of malignant neoplasms greatly increased expected years without and with activity limitation (2.0–2.8 and 1.0–1.2 years, respectively). Elimination of ischemic heart disease increased expected years without activity limitation (0.4–0.7 years), as did elimination of cerebrovascular diseases (1.0–1.1 years); however, there were only very small changes in years with activity limitation after eliminating these diseases (≤0.1 years). Elimination of the other 3 diseases and injuries slightly increased expected years without activity limitation (0.2–0.8 years) and slightly decreased years with activity limitation (0.1–0.8 years).

At birth, the expected years with non-ADL limitation and with ADL limitation were 4.6 and 3.8 years in males, respectively, and 5.9 and 5.9 years in females. Elimination of malignant neoplasms and ischemic heart disease increased expected years with ADL limitation (0.1–0.8 years). In contrast, elimination of cerebrovascular diseases, dementia, and fracture led to modest decreases (0.1–0.4 years). Elimination of shoulder lesions/low back pain very slightly decreased expected years with ADL limitation (0.0–0.1 years) and decreased years with non-ADL limitations (0.5–0.8 years).

## DISCUSSION

Elimination of malignant neoplasms, ischemic heart disease, and cerebrovascular diseases greatly increased life expectancy at birth, whereas elimination of dementia, shoulder lesions/low back pain, and fracture resulted in very small gains. These differences correspond to known disparities between fatal and nonfatal diseases and injuries.^1^^,^^8^^,^^15^ Elimination of nonfatal diseases and injuries, as well as elimination of fatal diseases, increased expected years of life without activity limitation. These findings were consistent with those of previous studies in several countries and confirmed that, in Japan, the effects of diseases and injuries on disability-free life expectancy differ considerably from those on total life expectancy.^1^^–^^4^^,^^18^

The results observed in the present study were due to the prevalence of activity limitation from diseases and injuries as well as death rates.^1^^,^^2^ As shown in Tables 1 and 2, individuals with cerebrovascular diseases or dementia had a high prevalence of low ADL.^3^^,^^4^^,^^19^ Therefore, elimination of these diseases decreased expected years with ADL limitation and increased years without activity limitation. Thus, improving prevention of these diseases would be likely to increase disability-free life expectancy and decrease expected years with severe disability. Although elimination of these diseases is unrealistic, these findings illustrate the current burden of selected diseases/injuries on disability-free life expectancy in Japan and provide considerable information for health planning against diseases and injuries.^1^^,^^4^^,^^6^^,^^20^

There were some limitations in the present study. We selected the abovementioned 6 diseases and injuries because malignant neoplasms, ischemic heart disease, and cerebrovascular diseases are the leading causes of death in Japan,^11^ because dementia, fracture, and cerebrovascular diseases are the primary reasons for residence in long-term elder care facilities,^14^ and because shoulder lesions/low back pain are the most frequently encountered medical conditions among outpatients in hospitals, clinics, and facilities of Japanese traditional massage, acupuncture, moxibustion, and judo-orthopedics.^12^ Treatment in acupuncture and moxibustion facilities, as well as in hospitals and clinics, is covered by the Japanese national health insurance system.^21^ Studies focusing on gains in disability-free life expectancy from eliminating other diseases and injuries would provide very useful additional information.

Underlying cause of death was used in the present analysis. If deaths indirectly caused by a disease were not considered, the overall effect of the disease on life expectancy would be underestimated.^15^^,^^22^ Underestimation of some diseases, including hypertension and diabetes mellitus, would be large, while underestimation of malignant neoplasms, ischemic heart disease, and cerebrovascular diseases (which were selected in the present study) would be relatively small.^23^ The problem of using underlying cause of death is a common one—even in official statistics—in studies of life expectancy after eliminating causes of deaths.^1^^,^^2^^,^^4^^,^^6^^,^^8^^,^^15^

In the present study, we analyzed the primary disease or injury of inpatients in hospitals and clinics and residents of long-term elder care facilities. This, too, might result in an underestimation of the overall effect of diseases and injuries on expected years with and without activity limitation. Inpatient data were obtained from the Patient Survey, and data on residents of long-term elder care facilities were obtained from the Survey of Institutions and Establishments for Long-term Care. These surveys include only the primary disease or injury of the inpatient/resident.^13^^,^^14^ However, underestimation is unlikely for outpatients because data on the presence or absence of all diseases and injuries were analyzed.^12^

In many persons, activity limitations are associated with 2 or more diseases or injuries. For example, if a patient with cerebrovascular disease sustains a fracture and subsequent ADL limitation, cerebrovascular disease might be selected as the primary reason for the ADL limitation. If such a selection occurred relatively frequently and only data on primary disease or injury were used, the effect of fracture on expected years with ADL limitation would be underestimated. Another possibility is that shoulder lesions/low back pain are more frequent in patients whose activity had been limited by other diseases or injuries. By not excluding the effect of such diseases or injuries, gains in years without activity limitation expected from eliminating shoulder lesions/low back pain would be overestimated.

Data on the disease status of outpatients were obtained from national health statistics, on the basis of responses from patients or their family members, and were not classified by ICD-10 code.^12^ The codes used for the response “shoulder lesions/low back pain” were M54.3–M54.5 and M75 in the present study, after referring to the classification of the Patient Survey. The responses to the survey and the codes we used might have been inaccurate. The effect of such errors on our results is unknown. Data for inpatients in hospitals and clinics and residents in long-term elder care facilities were based on the diagnoses of health care professionals, were classified by ICD-10 code, and are assumed to be highly accurate.^13^^,^^14^

The prevalence of activity limitation after eliminating a disease was calculated from the population excluding outpatients with the disease and activity limitation, inpatients with the disease in hospitals and clinics, and residents of long-term elder care facilities who had the disease. Persons with a condition of interest who were not receiving medical care were not considered in our study because most would not have been substantially affected by the condition. By not considering those persons, the effect of medical conditions of interest on expected years with activity limitation in the whole population would be slightly underestimated.

Gains in life expectancy at birth in Japan in 2007 after elimination of some diseases and injuries were reported in national official statistics.^8^ Those values, which were estimated using complicated approaches, were very similar to our estimates: 4.04 years for males and 3.01 years for females from elimination of malignant neoplasms, and 1.06 years for males and 1.15 years for females from elimination of cerebrovascular diseases (the values in Table 3 were 4.00, 2.98, 1.04, and 1.13 years, respectively).

The method we used to calculate years of life with and without activity limitation expected from elimination of diseases and injuries was proposed in 1983 and applied in several studies.^1^^,^^2^^,^^4^^,^^6^ As mentioned above, in this method, the use of life tables and prevalences of disability after eliminating diseases and injuries requires application of the Sullivan method. Although it is assumed that the age-specific prevalence of disability in a stationary population is equivalent to that observed in the real population, the Sullivan method is a common tool for estimating disability-free life expectancy based on cross-sectional data on disability.^17^ It would be helpful to use longitudinal data to estimate years gained from eliminating diseases and injuries.^1^^,^^3^^,^^5^

In conclusion, we estimated gains in years with and without activity limitation expected from elimination of selected diseases and injuries in Japan. Our results indicate that improving prevention of some of these diseases and injuries, including cerebrovascular diseases and dementia, might increase disability-free life expectancy and decrease expected years with severe disability.
